# Big Data and Network Biology 2015

**DOI:** 10.1155/2015/604623

**Published:** 2015-11-01

**Authors:** Shigehiko Kanaya, Md. Altaf-Ul-Amin, Samuel K. Kiboi, Farit Mochamad Afendi

**Affiliations:** ^1^Nara Institute of Science and Technology (NAIST), Nara 630-0192, Japan; ^2^University of Nairobi, Nairobi 00100, Kenya; ^3^Bogor Agricultural University, West Java 16680, Indonesia

Recently, biology has become a data intensive science because of huge datasets produced by high throughput molecular biological experiments in diverse areas including the fields of genomics, transcriptomics, proteomics, and metabolomics. In molecular biology, the list of components at the genome, transcriptome, proteome, and metabolome levels is gradually becoming complete and well-known to scientists. However, it is not holistically known how these components interact with each other to grow and maintain and reproduce life at different phases, in different environments, or with different challenging conditions. Networks at the molecular level are constructed to understand and explain processes and subprocesses of the cell. New tools and algorithms are being continuously developed for the purpose of handling and mining big biological data and networks aiming to serve humanity by developing smart health care systems, new generation medical tests, drugs, foods, fuel, materials, sensors, and so on. Overall, this improves the understanding of the cell or in other words the life as a system. Therefore, the range of topics under big data and network biology is extensive and the present special issue is not a comprehensive representation of the subject. Nonetheless, the articles selected for this special issue represent versatile topics concerning the title that we have the pleasure of sharing with the readers.

The review paper “A Glimpse to Background and Characteristics of Major Molecular Biological Networks” focuses on biological background and topological properties of gene regulatory, transcriptional regulatory, protein-protein interaction, and metabolic and signaling networks. Versatile information contained in this article is helpful to facilitate a comprehensive understanding and to conceptualize the foundation of network biology.

The paper titled “METSP: A Maximum-Entropy Classifier Based Text Mining Tool for Transporter-Substrate Identification with Semistructured Text” discusses a method for identifying transporter-substrate pairs by text mining and applied it to human transporter annotation sentences collected from UniProt database. The substrates of a transporter are not only useful for inferring function of the transporter, but also important in discovery of compound-compound interactions and reconstruction of metabolic pathways.

Volatile organic compounds (VOCs) play an important role in chemical ecology specifically in the biological interactions between organisms and ecosystems. The paper titled “Development and Mining of a Volatile Organic Compound Database” discusses creation of a new VOC database by collecting information scattered in scientific literature and analyzed the accumulated data to show relations between biological functions and chemical structures of VOCs. This work also shows that VOC based classification of microorganisms is consistent with their classification based on pathogenicity.

When inconsistent policies are applied to hospital computer systems, it can produce enormous problems, such as stolen information, frequent failures, and loss of the entire or part of the hospital data. The paper “EMRlog Method for Computer Security for Electronic Medical Records with Logic and Data Mining” presents a new method named EMRlog for computer security systems in hospitals based on two kinds of policies, that is, directive and implemented policies.

The paper “Cellular Metabolic Network Analysis: Discovering Important Reactions in* Treponema pallidum*” focuses on identifying critical reactions by analyzing the topological structure of the metabolic network.* Treponema pallidum* is the syphilis-causing pathogen and the critical reactions of its metabolism are important drug targets and such information can lead to invention of effective vaccine of syphilis.

The paper “Systematic Analysis of the Associations between Adverse Drug Reactions and Pathways” focuses on identifying relations between adverse drug reactions (ADRs) and biological pathways by integrating clinical phenotypic data, biological pathway data, and drug-target relations. This work suggests that drug perturbation in a certain pathway can cause changes in multiple organs, rather than in a specific organ.

The paper “Discovering Distinct Functional Modules of Specific Cancer Types Using Protein-Protein Interaction Networks” proposes a new graph theory based method to identify distinct functional modules associated with specific cancer types. The method was applied to nine different cancer PPI networks. The distinct modules identified by this work have a high correlation with those found in the experimental datasets related to specific cancer types.

The paper “Shaped 3D Singular Spectrum Analysis for Quantifying Gene Expression, with Application to the Early Zebrafish Embryo” presents a new approach for studying gene expression in nuclei located in a thick layer around a spherical surface by integrating several methods such as depth equalization on the sphere, flattening, interpolation to a regular grid, pattern extraction by shaped 3D singular spectrum analysis (SSA), and interpolation back to original nuclear positions.

The paper titled “Analysis of Chemical Properties of Edible and Medicinal Ginger by Metabolomics Approach” has investigated the chemical properties of medicinal and edible ginger cultivars using a liquid-chromatography mass spectrometry (LC-MS) approach. This work suggests the importance of acetylated derivatives of gingerol as medicinal indicators.

The paper titled “Development of Self-Compressing BLSOM for Comprehensive Analysis of Big Sequence Data” proposes a new and faster variant of self-organizing map (SOM) algorithm for comprehensive analysis of big sequence data without the use of high-performance super computers. The performance of the proposed method has been verified by applying it to bacterial genome sequences. The new approach has been able to cluster the sequences according to phylotype with high accuracy.

The paper “An Effective Big Data Supervised Imbalanced Classification Approach for Ortholog Detection in Related Yeast Species” proposes a supervised Pairwise Ortholog Detection (POD) approach by combining a set of gene pair features based on similarity measures, such as alignment scores, sequence length, gene membership to conserved regions, and physicochemical profiles. The performance of the proposed method has been compared with several existing methods in the context of three pairs of yeast genomes.

